# Comparison of staged-stent and stent-assisted coiling technique for ruptured saccular wide-necked intracranial aneurysms: Safety and efficacy based on a propensity score-matched cohort study

**DOI:** 10.3389/fneur.2023.1101859

**Published:** 2023-01-23

**Authors:** Guanghao Zhang, Renkun Zhang, Yanpeng Wei, Rundong Chen, Xiaoxi Zhang, Gaici Xue, Nan Lv, Guoli Duan, Chuanchuan Wang, Ying Yu, Dongwei Dai, Rui Zhao, Qiang Li, Yi Xu, Qinghai Huang, Pengfei Yang, Qiao Zuo, Jianmin Liu

**Affiliations:** Neurovascular Center, Changhai Hospital, Naval Medical University, Shanghai, China

**Keywords:** endovascular treatment, intracranial aneurysm, procedure-related complications, ruptured wide-necked aneurysm, acute subarachnoid hemorrhage (SAH), staged stent-assisted coiling

## Abstract

**Background:**

Application of stent-assisted coiling and FD in acute phase of ruptured wide-necked aneurysms is relatively contraindicated due to the potential risk of ischemic and hemorrhagic complications. Scheduled stenting after initial coiling has emerged as an alternative paradigm for ruptured wide-necked aneurysms. The objective of this study is to evaluate the safety and efficacy of a strategy of staged stent-assisted coiling in acutely ruptured saccular wide-necked intracranial aneurysms compared with conventional early stent-assisted coiling strategy *via* propensity score matching in a high-volume center.

**Methods:**

A retrospective review of patients with acutely ruptured saccular wide-necked intracranial aneurysms who underwent staged stent-assisted coiling or conventional stent-assisted coiling from November 2014 to November 2019 was performed. Perioperative procedure-related complications and clinical and angiographic follow-up outcomes were compared.

**Results:**

A total of 69 patients with staged stent-assisted coiling and 138 patients with conventional stent-assisted coiling were enrolled after 1:2 propensity score matching. The median interval time between previous coiling and later stenting was 4.0 weeks (range 3.5–7.5 weeks). No rebleeding occurred during the intervals. The rate of immediate complete occlusion was lower with initial coiling before scheduled stenting than with conventional stent-assisted coiling (21.7 vs. 60.9%), whereas comparable results were observed at follow-up (82.5 vs. 72.9%; *p* = 0.357). The clinical follow-up outcomes, overall procedure-related complications and procedure-related mortality between the two groups demonstrated no significant differences (*P* = 0.232, *P* = 0.089, *P* = 0.537, respectively). Multivariate analysis showed that modified Fisher grades (OR = 2.120, *P* = 0.041) were independent predictors for overall procedure-related complications and no significant predictors for hemorrhagic and ischemic complications.

**Conclusions:**

Staged stent-assisted coiling is a safe and effective treatment strategy for acutely ruptured saccular wide-necked intracranial aneurysms, with comparable complete occlusion rates, recurrence rates at follow-up and overall procedure-related complication rates compared with conventional stent-assisted coiling strategy. Staged stent-assisted coiling could be an alternative treatment option for selected ruptured intracranial aneurysms in the future.

## Introduction

Stent-assisted coiling (SAC) and Flow-diversion (FD) treatments have been demonstrated to be amenable paradigms for unruptured intracranial aneurysms with parent artery preservation (1–3). However, for the treatment of acutely ruptured wide-necked aneurysms, the deployment of stents and FD in the acute phase remains controversial (4, 5), and has 3 main issues. First, these device-implanted techniques may contribute to perioperative thromboembolic and hemorrhagic events due to the hypercoagulable status in the setting of subarachnoid hemorrhage (SAH) (6). Second, mandatory antiplatelet medication potentially increases the risk of symptomatic hemorrhagic complications from additional procedures (such as external ventricular drainage and craniotomy) and aggravates bleeding during the acute period (7, 8). Third, early cerebral vasospasm of SAH makes microwire navigation, microcatheter positioning, device distribution, and deployment challenging (9). For these issues, applications of SAC and FD are limited. Simultaneously, the option of protecting against rebleeding and accepting neck remnants instead of complete occlusion of the aneurysm in the acute phase has emerged, although it is controversial (10).

In these situations, the efficacy of the application of stents or FD with or without coils after initial coiling of acutely ruptured wide-neck intracranial aneurysms, so-called staged treatment, has been revealed recently (10–14). However, previous studies have included various subtypes of ruptured wide-necked aneurysms with different hemodynamic situations, angioarchitecture, and perioperative risk (such as saccular, fusiform, dissecting, pseudo-, and blood blister-like aneurysms), resulting in great heterogeneity in the safety and efficacy of this technique. Meanwhile, there is a lack of published reports directly comparing the safety and effectiveness profiles of conventional and staged treatment. Therefore, this study focused on staged stenting with or without additional coils after initial coiling of acute ruptured saccular wide-neck intracranial aneurysms and presented herein a propensity score-matched cohort study comparing staged stent-assisted coiling (s-SAC) with conventional SAC (c-SAC) in a high-volume center to further evaluate the safety and efficacy of staged stent placement for the treatment of acutely ruptured saccular wide-necked intracranial aneurysms.

## Methods

### Institutional experience

This retrospective, observational study conducted in line with the Strengthening the Reporting of Observational Studies in Epidemiology (STROBE) was approved by our local institutional review board (the Medical Ethics Committee of Changhai Hospital). Given the retrospective nature of the analysis, the requirement for written informed consent was waived.

### Patient selection and population

The inclusion criteria were as follows: (1) Spontaneous SAH diagnosed by CT or lumbar puncture and ruptured wide-necked aneurysms diagnosed by digital subtraction angiography (DSA); (2) dome-to-neck ratio <2 or a neck width of at least 4 mm measured by DSA; (3) aneurysm treated <3 d after the initial rupture; and (4) aneurysm treated by c-SAC or s-SAC technique. The exclusion criteria were as follows: (1) fusiform, traumatic, dissecting, pseudo-, and blood blister-like aneurysms; (2) parent artery occlusion, simple coiling or stent placement alone; (3) multiple aneurysms but failed to identify the ruptured one; and (4) incomplete clinical data.

**Figure 1:**
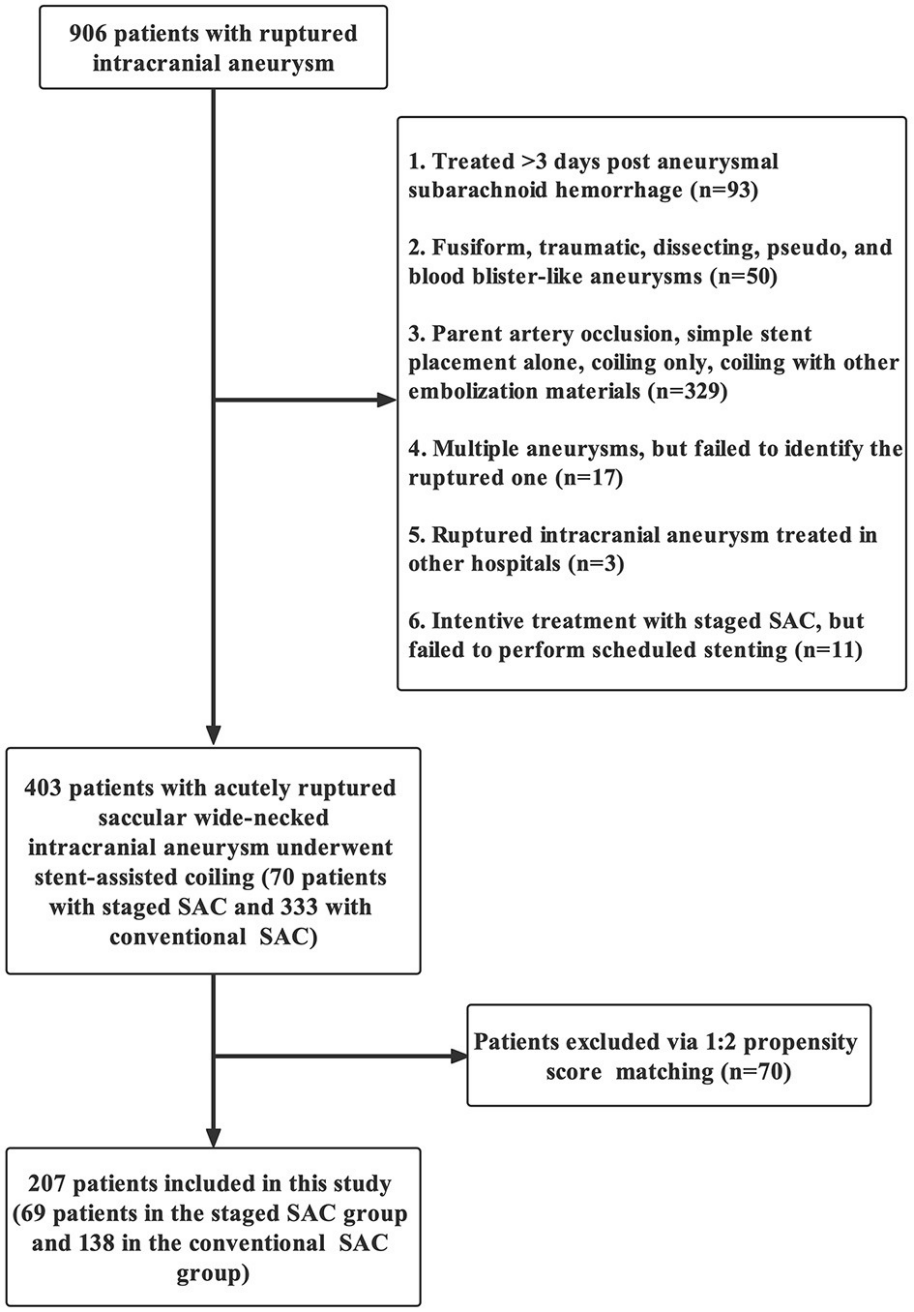
Flow diagram of patient selection according to the inclusion and exclusion criteria.

Clinical and angiographic data of 403 patients with ruptured intracranial aneurysms (RIA) from November 2014 to December 2019 were retrospectively reviewed by 2 experienced neurologists, including 70 patients who underwent s-SAC and 333 patients who underwent c-SAC. Propensity score matching (PSM) (1:2 matching) was used to adjust the potential differences in age, sex, hypertension, aneurysm location, aneurysm size, aneurysm neck, Hunt–Hess scale and stent type with a matching accuracy of 0.02. Finally, 69 and 138 propensity score-matched cases were included in this study, respectively (Figure 1).

### Endovascular procedure and medications

All procedures were performed *via* the femoral approach with the patient under general anesthesia by experienced endovascular neurosurgeons. Systemic heparinization was administered immediately after femoral sheath placement to maintain an activated clotting time of 2–3 times the baseline during the procedure. A 6F guiding catheter was placed into the distal internal carotid artery or vertebral artery. For accurate measurement, three-dimensional reconstruction was performed to assess the aneurysm and parent artery morphology. All stents (LVIS, MicroVention Terumo, USA; Enterprise, Cordis, USA; Solitaire, Covidien, USA; Neuroform, Boston Scientific, USA) and coils were deployed according to the standard procedure recommended by the manufacturer.

For the c-SAC group, in the acute phase, a loading dose of aspirin (300 mg) and clopidogrel (300 mg) was administered orally or rectally after stent placement. For the s-SAC group, in the acute phase, conventional coiling was performed without antiplatelet administration, the purpose of which was to embolize the ruptured site of the target aneurysm to avoid early rebleeding, then embolize as far as possible up to the neck remnant in the initial coiling.

Once patients neurologically recovered from the acute phase after SAH, stent implantation was scheduled after a required period of time (4 weeks). Thromboelastogram tests were performed in all patients in the s-SAC group on the day of scheduled admission for stenting. Dual antiplatelet drugs (aspirin 100 mg/day plus clopidogrel 75 mg/day or ticagrelor 180 mg/day according to whether the platelet response is adequate or not) were administered for at least 3 days before stenting. For both groups, dual antiplatelet drugs were recommended for 6 weeks post-procedure, followed by aspirin alone indefinitely.

### Clinical and angiographic outcomes

Clinical evaluations and follow-up assessments were performed by two experienced neurologists. The modified Rankin Scale (mRS) was retrospectively used to describe the extent of patient disability at the time of discharge from hospital and at clinical follow-up visits. Favorable clinical outcomes were defined as a mRS score of 0 to 2, and poor clinical outcomes were defined as a mRS score of 3 to 6. Angiographic follow-up was assessed by magnetic resonance angiography or DSA routinely at 6 months. After the procedure and yearly thereafter and was classified using the Raymond–Roy occlusion classification: Raymond 1 (complete occlusion), 2 (residual neck), and 3 (residual aneurysm).

### Statistical analysis

Statistical analysis was performed using R software (4.0.3). PSM was performed using MatchIt package to adjust the potential differences in age, sex, hypertension, aneurysm location, aneurysm size, aneurysm neck and Hunt-Hess scale. Continuous variables are expressed as mean values ± standard deviation (SD). Categorical variables are reported as proportions. And Pearson χ^2^ test, Fisher exact test, independent samples *t*-test, or non-parametric test was used for statistical, analysis as appropriate. A *P*-value < 0.05 was considered statistically significant. Univariate and multivariable analyses were performed to identify the association between procedure-related complications and predictive risk factors. The univariate analysis cutoff for inclusion in the multivariable analysis was *P* < 0.20. A *P*-value < 0.05 was considered statistically significant.

## Results

### Baseline characteristics

There were no statistically significant differences in all baseline characteristics after PSM between the two groups. Of the 207 patients, 89 (43%) patients were male. The mean age was 54.1 ± 10.7 years (range 33–88). The mean aneurysm size, aneurysm neck, and dome-to-neck ratio were 4.67 mm (IQR 3.4–6.0), 3.0 mm (IQR 2.2–4.0), and 1.63 (IQR 1.35–1.90), respectively. A total of 192 (92.75%) were in the anterior circulation, and 15 (7.25%) were in the posterior circulation. Clinical and demographic patient data before and after propensity score matching are summarized in Table 1.

**Table 1:** Angiographic and clinical characteristics before and after propensity score matching (*n* = 473).

**Characteristics**	**Total population**		***P*-value**	**Propensity score matching**		***P*-value**

	**s-SAC** **(*****n*** = **70)**	**c-SAC** **(*****n*** = **403)**		**s-SAC** **(*****n*** = **69)**	**c-SAC** **(*****n*** = **138)**	
Age, yrs	54.6 (9.6)	59.2 (11.8)	0.002	54.6 (9.6)	53.8 (11.2)	0.597
**Sex**						
Male	29 (41.4)	123 (30.5)	0.071	28 (40.6)	61 (44.2)	0.728
Female	41 (58.6)	280 (69.5)		41 (59.4)	77 (55.8)	
Hypertension, *n* (%)	46 (65.7)	212 (52.6)	0.042	45 (65.2)	81 (58.7)	0.450
Coronary heart disease, *n* (%)	3 (4.3)	20 (5.0)	1	3 (4.3)	6 (4.3)	1.000
Diabetes mellitus, *n* (%)	6 (8.6)	33 (8.2)	0.914	6 (8.7)	11 (8.0)	1.000
Smoking (%)	12(17.1)	40 (9.9)	0.075	11 (15.9)	21 (15.2)	1.000
Intraventricular hematoma (%)	20 (28.6)	150 (37.2)	0.164	20 (28.99)	43 (31.2)	0.873
Size [median (IQR)]	4.7 [3.4, 6.1]	4.5 [3.2, 6.2]	0.209	4.7 [3.4, 6.0]	4.3 [3.0, 6.6]	0.260
Neck [median (IQR)]	3.0 [2.2, 4.0]	3.3 [2.4, 4.3]	0.245	3.0 [2.2, 4.0]	3.0 [2.2, 4.0]	0.424
Dome to neck radio [median (IQR)]	1.6 [1.4, 1.9]	1.4 [1.1, 1.6]	0.001	1.6 [1.4, 1.9]	1.5 [1.3, 1.8]	0.160
**Location (%)**						
ICA	10 (14.3)	62 (15.4)	0.373	10 (14.5)	17 (12.3)	0.835
ACA	1 (1.4)	15 (3.7)		1 (1.5)	0 (0.0)	
AcomA	21 (30.0)	87 (21.6)		20 (29.0)	41 (29.7)	
MCA	12 (17.1)	49 (12.2)		12 (17.4)	23 (16.7)	
PcomA	22 (31.4)	156 (38.7)		22 (31.9)	46 (33.3)	
PC	4 (5.7)	34 (8.4)		4 (5.8)	11 (8.0)	
**Parent artery configuration**						
Bifurcation	40 (57.1)	212(52.6)	0.483	39 (56.5)	76 (55.1)	0.961
Side wall	30 (42.9)	191 (47.4)		30 (43.5)	62 (44.9)	
**Multiple aneurysms**						
Single	52 (74.3)	306 (75.9)	0.767	51 (73.9)	103 (74.6)	1.000
Multiple	18 (25.7)	97(24.1)		18 (26.1)	35(25.4)	
**Hunt-Hess (%)**						
1	35 (50.0)	202 (50.1)	0.779	34 (49.3)	67(48.6)	1.000
2	24 (34.3)	119 (29.5)		24 (34.8)	49(35.5)	
3	8 (11.4)	47 (11.7)		8 (11.6)	16(11.6)	
4	3 (4.3)	33 (8.2)		3 (4.4)	6(4.4)	
**Stent type (%)**						
LVIS	51(72.9)	279 (60.8)	0.312	51(73.9)	82(59.4)	0.053
Enterprise	17(24.3)	150 (32.8)		17(24.6)	45(32.6)	
Neuroform	2 (2.9)	25 (5.5)		1(1.5)	11(8.0)	
Solitaire	0 (0)	5 (1.0)		0 (0)	0 (0.0)	
**Modified Fisher grade (%)**						
1	11 (15.7)	78 (19.4)	0.423	11 (15.9)	25 (18.1)	0.789
2	43 (61.4)	241 (60.0)		42 (60.9)	88 (63.8)	
3	15 (21.4)	65 (16.2)		15 (21.7)	22 (15.9)	
4	1 (1.4)	18 (4.5)		1 (1.5)	3 (2.2)	
**Surgical procedure**						
EVD	9 (12.9)	20 (4.5)	0.012	8 (11.6)	5 (3.6)	0.054
Other	8 (11.4)	16 (3.5)	0.001	8 (11.6)	5 (3.6)	0.054

mm, millimeter; ICA, internal carotid artery; ACA, anterior cerebral artery; MCA, middle cerebral artery; ACoA, anterior communicating artery; PCoA, posterior communicating artery; PC, posterior circulation; EVD, external ventricular drainage.

Unless indicated otherwise, data are presented as the number of patients (%).

### Periprocedural complications

The rate of overall perioperative procedure-related complications in the s-SAC group was lower than that in the c-SAC group without statistical significance (4.3% vs. 8.9%, *P* = 0.394). Among the hemorrhagic complications, intraprocedural rupture, post-procedural early rebleeding and surgical procedure-related hemorrhagic events occurred in 1 case (1.4%), 0 case and 0 case in the s-SAC group, compared with 2 cases (1.4%), 2 cases (1.4%) and 3 cases (2.2%) in the c-SAC group (*P* = 1.000, 0.802, and 0.537), respectively. Among the ischemic complications, the rates of intraprocedural thrombosis and post-procedural thrombosis were comparable between the two groups (1.4 vs. 2.9%, *P* > 0.873; 0 vs. 1.4%, *P* = 0.802). One patient in the s-SAC group (1.4%, 1 of 69) suffered coil protrusion into the parent artery without clinical symptoms. The procedure-related mortality rate was 2.2% (3/138) in the c-SAC group, including 1 case of intraprocedural aneurysm rupture and 2 cases of post-procedural early rebleeding, compared with 0% in the s-SAC group (*P* = 0.537) (Table 2).

**Table 2:** Perioperative complications.

**Perioperative complications**	**Group**		***P*-value**

	**s-SAC** **(*****n*** = **69)**	**c-SAC** **(*****n*** = **138)**	
Procedure-related complications	3 (4.3)	12 (8.9)	0.394
**Hemorrhagic**	1 (1.4)	6 (4.3)	0.497
Intraprocedural rupture	1 (1.4)	2 (1.4)^*^	1.000
Aneurysm rebleeding	0 (0.0)	2 (1.4)^*^	0.802
Surgical procedure-related hemorrhagic event	0 (0.0)	3 (2.2)	0.537
**Ischemic**	1 (1.4)	6 (4.3)	0.497
Intraprocedural thrombosis	1 (1.4)	4 (2.9)	0.873
Post-procedural thrombosis	0 (0.00)	2 (1.4)	0.802
Coil protrusion	1 (1.4)	0 (0.00)	1.000
Salvage technique	0	0	1.000
Cerebral vasospasm	4 (5.8)	9 (6.5)	1.000
Procedure-related mortality	0 (0.00)	3 (2.2)	0.537

* One patient have two complications.

### Clinical and angiographic results

The immediate embolization results showed that in the s-SAC group following initial coiling, Raymond class I occlusion was achieved in 15 patients (21.7%), Raymond class II in 36 patients (52.2%), and Raymond class III in 18 patients (26.1%), compared with 84 patients (60.9%), 18 patients (13.0%), and 36 patients (26.1%) in the c-SAC group, respectively, demonstrating a statistically significant difference between the two groups (*P* < 0.001). For the s-SAC group, the median time between initial coiling and later stent treatment was 4.0 weeks (range 3.5–7.5 weeks). No rebleeding occurred during the intervals. Stents were implanted successfully in all 69 patients, resulting in 100% technical success. A total of 97.1% (67/69) of patients in the s-SAC group and 90.6% (125/138) of patients in the c-SAC group had favorable neurologic outcomes at discharge, and the difference between the two groups was not statistically significant (*P* = 0.155).

A total of 6 patients died at discharge in the two groups. In addition to the three patients who died of procedure-related complications mentioned above, the remaining three patients died of poor clinical grade at presentation and comorbidity. Therefore, a total of 201 patients survived at discharge. Among them, 188 patients (93.5%, 188 of 201) had been followed up clinically for 345–1,965 d (mean, 1,205 d). In addition, 61 patients (95.3%, 61/64) had favorable clinical outcomes in the s-SAC group, while 112 (90.3%, 112/124) patients had favorable clinical outcomes in the c-SAC group (*P* = 0.232).

**Figure 2:**
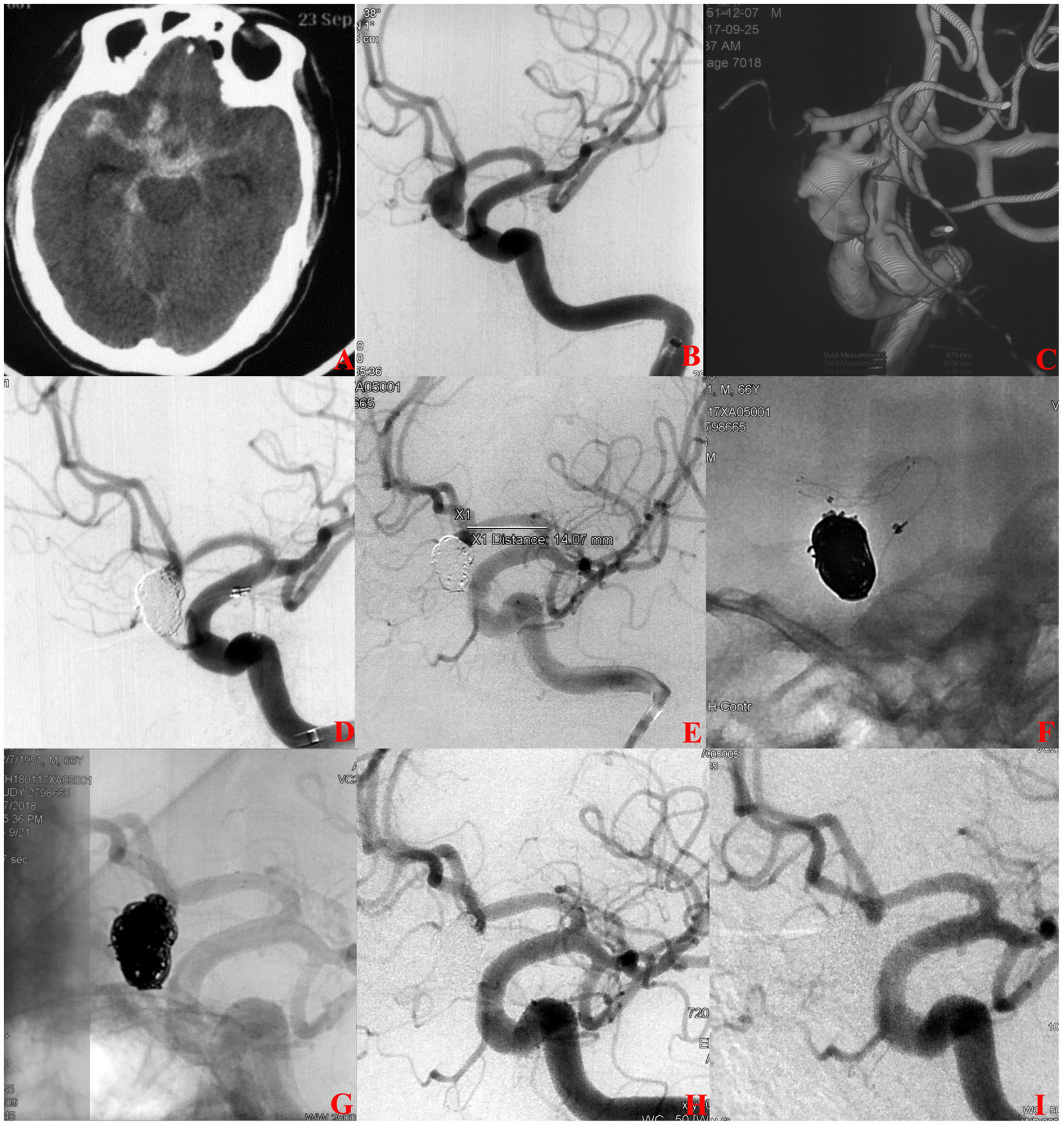
A ruptured anterior communicating aneurysms (AcomA) intracranial aneurysm treated with staged stent-assisted coiling. **(A)** The patient was admitted with spontaneous subarachnoid hemorrhage. **(B, C)** Cerebral angiography and 3D reconstruction revealed a AcomA aneurysm. **(D)** The aneurysm was treated with coiling embolization only at the initial stage. Immediate angiography showed the residual neck of the aneurysm. **(E–G)** 28 days later, the 3.5-mm * 15-mm LVIS stent adjunctive coils were deployed as scheduled. **(H)** Immediate angiography showed that the aneurysm was completely occluded. **(I)** Eleven months later, angiographic images showed complete occlusion of the aneurysm.

**Figure 3:**
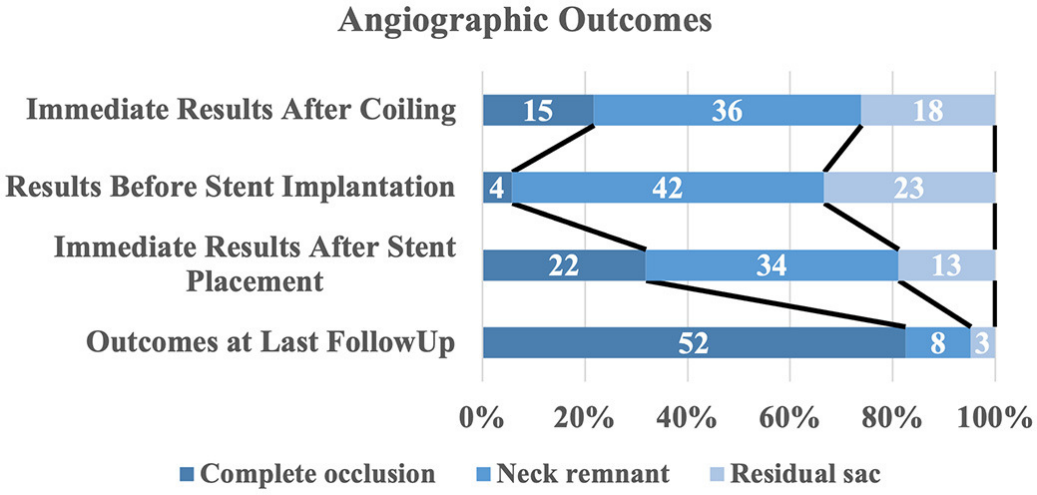
Distribution of angiographic outcomes by treatment stage. The raw distribution of results is shown.

A total of 188 (90.0%, 181/201) patients had at least one angiographic follow-up (mean 565 days), including 63 in the s-SAC group and 118 in the c-SAC group. Angiographic follow-up results showed that in the s-SAC group, 52 patients (82.5%, 52/63) were successfully occluded (Figure 2), 3 patients (4.8%, 3/63) improved, 6 patients (9.5%, 6/63) were stable, and 2 patients (3.2%, 2/63) were recanalized, compared with 86 patients (72.9%, 86/118), 8 patients (6.8%, 8/118), 12 patients(10.2%, 12/118), and 12 patients (10.2%, 12/118) in the c-SAC group, showing no statistically significant difference between the two groups (*P* = 0.357) (Table 3). The aneurysm occlusion rates in the s-SAC group including immediate results after coiling before stent implantation, immediate results after stent implantation and the outcomes at the last follow-up are summarized in Figure 3.

**Table 3:** Angiographic and clinical Outcomes.

**Outcomes**	**Group**		***P*-value**

	**s-SAC** **(*****n*** = **69)**	**c-SAC** **(*****n*** = **138)**	
**Angiographic outcome**			
**Immediate embolization**			
Raymond class I	15 (21.7)	84 (60.9)	<0.001
Raymond class II	36 (52.2)	18 (13.0)	
Raymond class III	18 (26.1)	36 (26.1)	
**Before stent implantation**			
Raymond class I	4 (5.8)		
Raymond class II	42 (60.9)		NA
Raymond class III	23 (33.3)		
**After stent implantation**			
Raymond class I	22 (31.9)		
Raymond class II	34 (49.3)		NA
Raymond class III	13 (18.8)		
**Follow-up**			
Complete occlusion	52 (82.5)	86 (72.9)	0.357
Improvement	3 (4.8)	8 (6.8)	
Stability	6 (9.5)	12 (10.2)	
Recurrence	2 (3.2)	12 (10.2)	
Retreatment	1 (1.6)	6 (5.2)	0.424
**Clinical outcome at discharge**			
0–2	67 (97.1)	125 (90.6)	0.155
3–6	2 (2.9)	13 (9.4)	
**Clinical follow-up** ^a^			
0–2	61 (95.3)	112 (90.3)	0.232
3–6	3 (4.7)	12 (9.7)	
**Clinical follow-up** ^b^			
0–2	61 (92.4)	112 (87.5)	0.296
3–6	5 (7.6)	16 (12.5)	

a Excluding patients who died at discharge.

b Including patients who died at discharge.

### Univariate and multivariate analysis of risk factors for perioperative procedure-related complications

The following factors were included in the univariate analysis of perioperative procedure-related complications: patient age, sex, history of hypertension, smoking history, history of diabetes, history of coronary heart disease, Hunt-Hess grade, modified Fisher grade, aneurysm size, neck size, dome-to-neck ratio, aneurysm location, treatment strategy, stent type, and immediate embolization results. Univariate analysis showed that modified Fisher grade (*P* = 0.025), bifurcation (0.044) and a history of diabetes (*P* = 0.043) were associated with overall procedure-related complications; neck size (*P* = 0.038) and a history of diabetes (*P* = 0.013) were associated with ischemic complications; modified Fisher grade and dome-to-neck ratio were associated with hemorrhagic complications. Multivariate analysis showed that modified Fisher grade (OR = 2.120, 95% CI 1.036–4.440 *P* = 0.041) was an independent predictor of overall procedure-related complications, while there were no predictors for the hemorrhagic and ischemic procedure-related complications.

## Discussion

In this propensity score-matched cohort study, the rates of overall procedure-related complications were slightly lower in the s-SAC group than in c-SAC group, while the differences were not statistically significant (*P* = 0.394). The angiographic follow-up results showed that the s-SAC group had a higher occlusion rate and lower recurrence rate than the early stent group, but the difference was not statistically significant. In addition, the rates of favorable clinical outcomes at discharge and during long-term follow-up were comparable between the two groups. Multivariate analysis revealed that c-SAC was an independent predictor of overall procedure-related complications. These results suggest that s-SAC has lower perioperative complication rates and comparable long-term angiographic outcomes when compared with the c-SAC strategy.

Despite evidence from prior research suggesting that conventional SAC for treating certain patients with intracranial aneurysms may be safe (15, 16), SAC of saccular wide-necked aneurysms is still controversial due to the uncertain incidence of procedure-related complications (17). Mandatory dual antiplatelet medication in the setting of acutely ruptured aneurysms increases the theoretical risk of hemorrhagic complications. A multicenter retrospective cohort confirmed this concern (18). The authors reported that the aneurysm rebleeding rate in the SAC group was significantly higher than that of coiling only group (17.4 vs. 1.9%, *P* < 0.007). Another 2 retrospective analyses suggested that antiplatelet therapy during SAH was associated with the risk of external ventricular drainage-related hemorrhagic complications (19, 20). Additionally, the potential risk of in-stent thrombosis makes conventional SAC disadvantageous due to the hypercoagulation condition in the acute phase of SAH. Our previous meta-analysis indicated that the thrombosis rate in the SAC group was significantly higher than that of the coiling-only group for RIA treatment (29.9 vs. 17.5%; RR = 2.71; 1.95–3.75) (21). A study with 55 cases of SAC and 394 cases of coiling alone for the treatment of acutely RIA without antiplatelet premedication showed that antiplatelet premedication-free SAC increased the risk of thromboembolism compared with coiling alone (22). Moreover, it is worth noting that cerebral vasospasm induced by acute SAH may present an extra obstacle when attempting to perform conventional SAC treatment. The structural thinness of the parent or branch arteries caused by cerebral vasospasm can have a negative impact on the navigation and delivery of stent catheters during procedures, which may lead to a decline in the technical success rate, stent migration and poor stent tolerance. In this study, for early coiling treatment before scheduled stenting, we did not use any antiplatelet therapy and relatively complex intravascular manipulation during the acute period of SAH and were more prone to accept a neck remnant if we consider that the primary purpose of early RIA management is to prevent rebleeding. Thus, theoretically, the incidence of procedure-related hemorrhagic events and thromboembolism can be reduced.

There were several studies implying that the s-SAC paradigm may be a favorable alternative in RIA treatment. Feng et al. reported 47 patients of s-SAC for acute ruptured wide-necked intracranial aneurysms and found that no hemorrhagic and ischemic complication was observed, and all patients demonstrated favorable clinical outcomes (mRS 0-2) at follow-up (11). Mine et al. evaluated the same strategy in 23 cases. No rebleeding occurred during the mean delay of 24.3 days between the initial coiling and stenting and clinical status was unchanged in all patients (10). However, the near-excellent results need additional case-control and larger sample studies to confirm the current observations. Our retrospective propensity score-matched cohort study suggested that the s-SAC treatment seems to be associated with a decreased risk of overall perioperative procedure-related complications compared with c-SAC treatment without statistical significance (4.3% vs. 8.9%, *P* = 0.394). The hemorrhagic and ischemic complications rates were lower in the s-SAC group than that in the c-SAC group, although the differences were not significant statistically. Notably, a total of 26 patients were treated with surgical procedures before or after endovascular treatment during SAH acute phase and we observed that the rate of hemorrhagic complications associated with surgical procedures in the c-SAC group was higher than that in the s-SAC group (3/10 vs. 0/16; 30.0 vs. 0%, *p* = 0.046). On the other hand, long-term clinical outcome was comparable between the two groups (*p* = 0.272). Our study results may be considered evidence of s-SAC treatment being a safe paradigm in this setting.

In terms of occlusion rates at follow-up, the s-SAC group yielded higher rates of complete occlusion and lower recurrence than the c-SAC group (82.54 vs. 72.88% and 3.17 vs. 10.17%, respectively). The long-term stability of s-SAC appeared to be superior to that of c-SAC, even though the difference did not meet the criteria for statistical significance (*P* = 0.357). We believe a complete obliteration rate of 82.54% in the s-SAC group to be rather satisfying and comparable with those reported in the previous research (10, 11). However, because of the lack of similar research in terms of study design and case scenario, poor comparability existed in the outcomes of different cohorts. Therefore, additional comparison studies are required to assess the safety and efficacy of the two SAC strategies for the treatment of acutely ruptured wide-necked intracranial aneurysms.

Since it is difficult to achieve complete occlusion of the wide-necked aneurysm using coiling alone without stent assistance, most neuroradiologists are concerned about early rebleeding during the interval between initial and complementary treatment due to the cerebral aneurysm rerupture after treatment study indicating that the degree of aneurysmal occlusion was strongly related to rerupture (23). However, recent studies have led to controversy regarding the feasibility of total aneurysm occlusion in the acute phase of SAH. Brinjikji et al. reported only one early rebleeding (3.22%) without additional morbidity in a cohort of 31 patients treated with complementary flow diverters (13). Recent literature on staged treatments of RIAs with scheduled implantation of stents or various approaches observed no early rebleeding (10–12). In the current study, our results further confirm this observation. For initial coiling treatment, our primary goal is to achieve enough packing density at the most likely rupture point and embolize as far as possible up to the neck remnant in the initial coiling (51/69, 73.91%). The immediate embolization outcome of initial coiling achieved Raymond I and Raymond II was comparable with the conversational SAC group (104/138, 75.36%, *P* = 0.821). The previous study has demonstrated that ruptured aneurysms with coiling only could be decreased to 2.0% with neck remnant occlusion. Based on the above facts, we consider the technique safe and effective for preventing early rebleeding.

The interval time between the initial coiling and scheduled SAC may be significant. The previous studies have demonstrated that the incidence of early rebleeding within 30 days after coiling of a saccular RIA is very low, ranging from 1.9 to 3.6% (24–26). And data on antiplatelet management for stent-assisted coiling/flow diversion in the acute stage of ruptured intracranial aneurysms are relatively scarce. It is difficult to provide neurosurgeons with practical guidance based on the limited data available, and the availability and accessibility of certain antiplatelet agents vary depending on the country/region. We consider that without antiplatelet administration during this period, there is no need to weigh the risk of thrombotic complications against the risk of rebleeding caused by inadequate antiplatelet drugs. Therefore, we set the time interval between initial coiling and scheduled stenting as 4 weeks, which is consistent with the previous studies (11, 12). However, there is no consensus on this issue due to the scarcity of data. Further exploration is still needed in this field.

Alternately, temporary stent-assisted coil embolization (coiling assisted by temporary stenting, CATS) and intra-aneurysmal flow disruption (IAFD) paradigms have been proposed, with the benefit that blood flow is not disrupted during treatment and no implants are left in the parent channel (27, 28). Since the first report on CATS in 2013, few reports have been published about the safety and effectiveness of this technique for RIA. A retrospective study of CATS using Comaneci device (Rapid Medical, Israel) for 118 saccular wide-necked RIA suggested that the technical success rate was 100% and 66.9% (75 of 112) and 8.73% (11 of 118) of the patients demonstrated favorable complete occlusion at follow-up and procedure-related complications (29). IAFD was specifically designed to treat wide-necked bifurcation aneurysms (28). According to a recent retrospective case-control study of patients with RIA treated with the IAFD or conventional coiling, IAFD yielded a similar procedural complication rate (19.2 vs. 22.7%, *P* = 0.447) and potentially improved angiographic outcome at follow-up (93.9 vs. 76.2% *P* = 0.058) (30). Although in our current study, compared with these studies, s-SAC also showed satisfactory complete occlusion at follow-up (82.5%) and procedure-related complications (4.3%), methodological and study design differences between the two studies limit comparability.

## Limitations

The present study has some limitations. First, the retrospective nature may have resulted in selection bias. In addition, we did not include other therapeutic devices and techniques in this study, particularly staged FD and surgical clipping. Therefore, it is difficult to determine if our treatment strategy would be superior to other treatment strategies in addition to conventional SAC. Lastly, due to the limited number of aneurysms in each location as a result of the small sample size, confirmation of our findings requires a large prospective study.

## Conclusion

s-SAC is a safe and effective treatment strategy for acutely ruptured saccular wide-necked intracranial aneurysms, with comparable complete occlusion rates, recurrence rates at follow-up, and overall procedure-related complication rates compared with the c-SAC strategy. s-SAC could be an alternative treatment option for selected RIA in the future. Prospective studies with larger sample sizes are required to further determine the safety and efficacy of this strategy.

## Data availability statement

The original contributions presented in the study are included in the article/supplementary material, further inquiries can be directed to the corresponding authors.

## Ethics statement

The studies involving human participants were reviewed and approved by the Medical Ethics Committee of Changhai Hospital. Written informed consent from the patients/participants or patients/participants' legal guardian/next of kin was not required to participate in this study in accordance with the national legislation and the institutional requirements.

## Author contributions

GZ, RZhan, and YW made substantial contributions to the conception and design, acquisition of data, analysis, and drafting of the manuscript. QZ, JL, NL, GD, XZ, QL, RZhao, YX, QH, and PY assisted in the evaluation of analysis and their interpretation. All authors read and approved the final manuscript.
